# Focus on Quantum Crystallography

**DOI:** 10.1107/S2052252525008759

**Published:** 2025-10-27

**Authors:** Paulina M. Dominiak, Ángel Martín Pendás, Krzysztof Woźniak

**Affiliations:** a University of Warsaw Biological and Chemical Research Centre, Faculty of Chemistry Żwirki i Wigury 101 Warszawa02-089 Poland; b Departmento Química Física y Analítica Universidad de Oviedo Oviedo33006 Spain

**Keywords:** quantum crystallography, chemical bonding, structure determination, materials science, drug discovery, theoretical chemistry, computational chemistry

## Abstract

This editorial introduces the ‘Focus on Quantum Crystallography’ collection, coinciding with the centenary of quantum mechanics and highlighting the convergence of crystallography and quantum theory. It outlines recent advances and applications of quantum crystallography in areas such as chemical bonding, materials science, drug discovery and theoretical chemistry. It showcases the growing role of quantum crystallography in bridging experimentation and computation.

The progress of civilization and technology is based on the development of science, which depends on the quality of data and the methods used to transform them into scientific laws or to validate newly proposed theories.

In 2025 we celebrate the centenary of Erwin Schrödinger’s development of wave mechanics and Werner Heisenberg’s, Max Born’s and Pascual Jordan’s proposal of matrix mechanics. This was the beginning of the era of Quantum Mechanics, whose history is inextricably intertwined with the growth of Crystallography since the first X-ray diffraction experiments on crystals were carried out.

The successful marriage of modern crystallography and quantum mechanics is based on the fact that the former requires quantum mechanical models to refine crystal structures, while the latter very often demands crystal structures as a starting point for the extensive quantum mechanical analyses needed to understand and predict the properties of the advanced materials on which our civilization depends.

It is not surprising, therefore, that Quantum Crystallography (QCr), situated at the intersection of Quantum Mechanics and Crystallography, is a burgeoning field that promises to bridge the gap between theory and experiment in understanding the fundamental behavior of matter at the atomic and molecular level.

It is an incomprehensible paradox that more than a century after the introduction of the first scattering model in crystallography, the independent atom model (IAM), more than 99% of all crystal structures (about 2 million) are still refined using it. In contrast to the major investments in improving the quality of the measured data, the resources employed to improve the methods used to interpret them are still scarce. In a sense, the overwhelming success of the IAM has slowed the development and application of new methodologies, *i.e.* new models of atomic electron densities, in refining structures. These new methodologies, which are being actively developed in QCr beyond the IAM, allow for the determination of accurate and precise charge and spin electron density distributions in position and momentum space from diffraction and scattering experiments. These methodologies also provide a wealth of parameters that are used to characterize the nature of chemical bonding or quantify physicochemical properties.

The current development of Quantum Crystallography goes far beyond the original definition given by Massa* et al.* (1995 ▸). According to this, the measured intensities of reflections can be used to extract the wavefunctions or density matrices of a system directly, and quantum mechanical calculations can also be used to enhance the accuracy of the crystallographic refinement. However, as is the case in the evolution of most scientific disciplines, this traditional statement does not embrace many newly proposed applications of QCr, and other definitions are circulating both in the scientific literature and at various scientific conferences. It is thus time to show these new advances.

In this cross-journal focused issue, in which a collection of articles from *IUCrJ*, *Acta Crystallographica* Sections A and B, and *Journal of Applied Crystallography* are brought together at https://journals.iucr.org/special_issues/2025/QCr/, we would like to show the dynamic development of QCr, its thematic richness and possible relations with some complementary fields. We hope that this will enable the identification of the main directions in which the quantum-crystallographic scientific revolution is evolving. Looking back, it is difficult to understand why it is only now that quantum crystallography is developing so intensively. We have no doubt that the potential of quantum crystallography is enormous and will change the face of many fields of contemporary and future science.

The fields that benefit from the development of QCr and are covered in this issue are presented in Table 1 ▸ and are summarized below.

(*a*) *Determination of the accurate geometry of complex molecular structures*. By coupling quantum mechanical calculations with experimental techniques, QCr enables researchers to overcome traditional barriers in both fields. For example, QCr provides accurate hydrogen-atom positions from conventional X-ray diffraction patterns with a level of precision similar to that obtained by neutron diffraction. QCr also improves atomic displacement parameters with a focus on their future use in the assessment of the thermodynamic properties of crystals and the probing of the local structure of materials.

(*b*) *Understanding chemical bonding beyond simple geometric analysis*. By revealing charge distributions, bond paths and subtle electronic effects that traditional, geometry-based crystallography cannot capture, QCr uncovers the nature of weak interactions, stronger atom–atom chemical bonding and electron delocalization phenomena. QCr also reveals complementarity in the electrostatic potential, offering a deeper, more accurate picture of bonding in molecules and crystals. For bonding information to be derived from experimental data, the quality of the data and improvements in data processing are of high importance.

(*c*) *Materials science*. Understanding the microscopic structure of materials is of paramount importance to developing new materials with tailored properties. Since the output of QCr is not just structural determination (atomic structures), but a wealth of electronic and bonding information, often embedded in machine-learned tools, new insights that facilitate the design of materials for a variety of applications, from electronics to pharmaceuticals, are provided.

(*d*) *Drug discovery*. In pharmaceutical research, knowledge of the energy landscape associated with structural determination is essential for rational drug design. QCr techniques are capable of accurately revealing and quantifying the interactions underlying the crystal packing observed in the case of polymorphs, and promise to be very useful in revealing the nature of drug–receptor interactions, guiding the development of more effective and targeted therapies. Basic research in the structural biology field could also benefit from QCr techniques, including coupling of improved modeling of electrostatic potential and charge density evaluation based on single-particle cryo-EM or 3D electron diffraction data.

(*e*) *Theoretical and computational chemistry*. Quantum crystallography synergizes with theoretical chemistry, providing experimental validation for computational models and methodologies. This interdisciplinary collaboration improves the accuracy and predictive power of computational chemistry approaches. Subdisciplines of QCr such as the X-ray restrained wavefunction (XRW) method promise to help theoreticians to propose better density functional approximations or examine scars in wavefunctions, or electron densities, of strong correlations that currently escape theoretical manipulation. Applications or proposals that are well known in theory but not in experiment, or vice versa, and that could thus benefit from the QCr interplay between both fields are presented.

The issue also includes two reviews covering research from the QCr field published over the last decade (Krawczuk & Genoni, 2025 ▸; Hoser & Madsen, 2025 ▸) as well as a historical perspective on QCr given by Cooper (2025 ▸).

As a final perspective, quantum crystallography is beginning to show how far structural science can go. By mapping electron densities with unmatched precision, it extends beyond the reach of traditional diffraction and opens new opportunities across disciplines. From clarifying molecular interactions in drug discovery to guiding the design of superconductors, quantum devices and catalysts for clean energy, its applications are wide ranging. Step by step, it is shaping into a versatile structure–function mapping tool – linking physics, chemistry, biology and materials science – and offering a practical path toward discoveries that can impact both technology and everyday life.

## Figures and Tables

**Table:** (*a*) Accurate atomic models

Using effective core potentials with ZORA in HAR accelerates refinement of heavy-element structures up to twofold without accuracy loss	*The use of effective core potentials in Hirshfeld atom refinement: making quantum crystallography faster in NoSpherA2* [Kleemiss, F., Meurer, F., Shenderovich, I. G. & Bodensteiner, M. (2025). *J. Appl. Cryst.***58**, 374–382]
expHAR, a new exponential Hirshfeld partition scheme, improves hydrogen ADPs and *X*—hydrogen bond lengths in HAR, outperforming conventional approaches	*Towards improved accuracy of Hirshfeld atom refinement with an alternative electron density partition* [Chodkiewicz, M. & Woźniak, K. (2025). *IUCrJ*, **12**, 74–87]
*ReCrystal* employs periodic DFT-derived multipoles for tailored TAAM, refining crystals with improved hydrogen accuracy and no external libraries	*Solid-state calculations for iterative refinement in quantum crystallography using the multipole model* [Patzer, M. & Lehmann, C. W. (2025). *IUCrJ*, **12**, 322–333]
HAR applied for the first time to electron diffraction, here for ice I_h_, shows limited benefit; accurate bond lengths require modeling dynamical scattering effects	*Hirshfeld atom refinement and dynamical refinement of hexagonal ice structure from electron diffraction data* [Chodkiewicz, M. L., Olech, B., Jha, K. K., Dominiak, P. M. & Woźniak, K. (2024). *IUCrJ*, **11**, 730–736]
Restraining hydrogen ADPs in HAR shows minimal effect on *X*—hydrogen bond lengths – which remain close to neutron values – but improved refinement stability	*Role of restraints on hydrogen atoms in Hirshfeld Atom Refinement: the case of tri-aspartic acid trihydrate* [Sankolli, R., Malaspina, L. A., Dolomanov, O. V., Luger, P., Holstein, J. J., Paulmann, C., Morgenroth, W., Kleemiss, F., Dittrich, B. & Grabowsky, S. (2025). *Acta Cryst.* B**81**, 484–497]
AAM_NoMoRe integrates HAR/TAAM with DFT-based methods to refine normal mode frequencies, improving hydrogen ADPs and enabling thermo­dynamic property estimation from X-ray diffraction	*Advancing dynamic quantum crystallography: enhanced models for accurate structures and thermodynamic properties* [Butkiewicz, H., Chodkiewicz, M., Madsen, A. Ø. & Hoser, A. A. (2025). *IUCrJ*, **12**, 123–136]
HAR with Gram–Charlier formalism on β-1,3-diacetylpyrene reveals anharmonic ADPs from routine X-ray data, underscoring thermal motion modeling	*Tracking anharmonic oscillations in the structure of β-1,3-di­acetylpyrene* [Zwolenik, A. & Makal, A. (2025). *IUCrJ*, **12**, 23–35]. Commentary: *Connecting lattice and molecular vibrations to organic crystal properties* [Catalano, L. (2025). *IUCrJ*, **12**, 6–7]
Benchmarking in-house X-ray diffractometers for 3D-ΔPDF shows that they capture local structural disorder, offering complementary data for future QCr approaches	*Benchmarking 3D-ΔPDF analysis using in-house X-ray sources* [Juul, K. O. R., Støckler, K. A. H. & Iversen, B. B. (2025). *Acta Cryst.* A**81**, 254–268]

**Table:** (*b*) Chemical bonding beyond geometry

Multipolar refinement and HAR accurately reproduce geometry and electron density of an iron(II) complex, validating chemical bonding analysis through charge density and topological studies	*Multipolar model and Hirshfeld atom refinement of tetra­aqua­bis­(hydrogenmaleato)iron(II)* [Ferreira Guimarães, H. & Lages Rodrigues, B. (2025). *Acta Cryst.* B**81**, 350–362]
Quantum crystallography critically depends on experimental data quality; high-quality structure factors of L-alanine and taurine enable reliable electron density and bonding analyses	*Accurate temperature dependence of structure factors of L-alanine and taurine for quantum crystallography* [Hayashi, M., Nishioka, T., Kasai, H. & Nishibori, E. (2025). *IUCrJ*, **12**, 384–392]
An empirical three-parameter absorption correction improves QCBED accuracy, separating local/non-local effects and enhancing unfiltered differential pattern matching	*A refinable three-parameter equation for phenomenological absorption in quantitative electron microscopy – determining the equation* [Nakashima, P. N. H., Liu, T., Smith, A. E. & Bourgeois, L. (2025). *J. Appl. Cryst.***58**, 1665–1676]
QTAIM, IQA and source function analyses of pyridine–halogen complexes reveal exchange–correlation rival electrostatics, challenging simplistic donor–acceptor bonding views	*The nature of halogen bonding: insights from interacting quantum atoms and source function studies* [Pisati, A., Forni, A., Pieraccini, S. & Sironi, M. (2025). *IUCrJ*, **12**, 188–197]
Electrostatic potential analysis links supramolecular motifs to the complementarity of charge concentration and depletion sites, generalizing graph-set theory and guiding future crystal engineering	*The origin of synthons and supramolecular motifs: beyond atoms and functional groups* [Shukla, R., Aubert, E., Brezgunova, M., Lebègue, S., Fourmigué, M. & Espinosa, E. (2025). *IUCrJ*, **12**, 334–357]. Commentary: *Electrostatic landscapes in crystal engineering: a new perspective on synthons* [Novikov, A. S. (2025). *IUCrJ*, **12**, 255–256]
Multipolar refinement, HAR and X-ray wavefunction fitting reveal ylid-type S—C bonding in WYLID, clarifying YLID bonding and carbonyl–enolate distinction.	*Synthesis and quantum crystallographic evaluation of WYLID: YLID’s red rival* [Meurer, F., Schimpf, M., Hischa, B., Hennig, C., Rehbein, J., Kleemiss, F. & Bodensteiner, M. (2025). *J. Appl. Cryst.***58**, 678–687]

**Table:** (*c*) Materials science

Periodic DFT yields elastic, piezooptic and photoelastic tensors, and identifies BaWO_4_ and PbWO_4_ as promising optoelectronic materials through predictive analysis	*Photoelasticity of crystals with the scheelite structure: quantum mechanical calculations* [Demyanyshyn, N. M., Mytsyk, B. G., Andrushchak, A. S. & Kityk, A. V. (2025). *Acta Cryst.* B**81**, 47–54]
Machine-learned FFLUX force field from QCT enables efficient ice polymorph simulations, predicting a new II′ phase and supporting polymorph screening	*A computationally efficient quasi-harmonic study of ice polymorphs using the FFLUX force field* [Pák, A., Brown, M. L. & Popelier, P. L. A. (2025). *Acta Cryst.* A**81**, 36–48]
QTAIM-based topological coordination number refines coordination analysis beyond traditional Voronoi–Dirichlet partitioning, enabling meaningful intermetallic bonding insights and supporting AI-driven structure–property prediction	*Topological coordination numbers and coordination reciprocity from electron-density distributions* [Wagner, F. R., Freccero, R. & Grin, Y. (2025). *Acta Cryst.* A**81**, 221–244]
Theoretical QCr reveals the nature of the pressure-induced *s* → *d* transition in cubic calcium, which shows multi-centered bonds and electride character with interstitial electron localization	*Looking at high-pressure electrides through the lens of quantum crystallography: the case of simple cubic calcium* [Racioppi, S. & Zurek, E. (2025). *Acta Cryst.* B**81**, 256–265]
Bonding theories applied to layered hybrid perovskites reveal structure–property relationships, phase transitions, and intermolecular interactions guiding multifunctional material design	*Effect of the transition metal on the structure and order–disorder phase transition in layered hybrid metal halides (CH_3_CH_2_NH_3_)_2_[*M*Cl_4_] (*M* = Mn and Co)* [Jakhi, S. S., Dhanetwal, M., Reddy, V. R. & Hathwar, V. R. (2025). *Acta Cryst.* B**81**, 363–372]
Experimental and theoretical QCr reveal halide-mediated Ni^2+^ magnetic exchange, with iodine showing weak covalency and through-space interactions enhancing magnetism	*Electron-density analysis of halide⋯halide through-space magnetic exchange* [Scatena, R., Manson, Z. E., Villa, D. Y., Manson, J. L., Allan, D. R., Goddard, P. A. & Johnson, R. D. (2025). *J. Appl. Cryst.***58**, 363–373]

**Table:** (*d*) Drug discovery and structural biology

Experimental and theoretical QCr of tyramine polymorphs reveal crystallization-condition effects on stability, interactions and pharmaceutical formulation design	*Additive-driven microwave crystallization of tyramine polymorphs and salts: a quantum crystallography perspective* [Grabowski, S., Nowakowska, K., Butkiewicz, H., Hoser, A., Wesełucha-Birczyńska, A., Seidler, T., Moskal, P. & Gryl, M. (2025). *IUCrJ*, **12**, 403–416]
Quantum crystallography of NSAID–COX binding reveals electrostatic determinants of selectivity, identifying key residues and guiding rational drug design	*Understanding the selectivity of nonsteroidal anti-inflammatory drugs for cyclooxygenases using quantum crystallography and electrostatic interaction energy* [Pawlędzio, S., Ziemniak, M., Wang, X., Woźniak, K. & Malinska, M. (2025). *IUCrJ*, **12**, 208–222]
Theoretical QCr with GruPol shows ionic strength modulates glucagon dipole moments and polarizabilities, predicting biomolecular electrical properties for drug delivery	*Electric charge and salting in/out effects on glucagon’s dipole moments and polarizabilities using the GruPol database* [Ligorio, R. F., Gehle, R. H. M., Dos Santos, L. H. R. & Krawczuk, A. (2025). *Acta Cryst.* B**81**, 192–201]
Applying TAAM in QCr to experimental biomacromolecular data highlights charged moieties at low resolution, stressing the importance of accurate scattering factors	*Protein electrostatic potential Fourier maps calculated using the transferable aspherical atom model and the independent atom model across resolutions* [Kulik, M. & Dominiak, P. M. (2025). *IUCrJ*, **12**, 616–632]

**Table:** (*e*) Theoretical and computational chemistry

Experimental benchmarking shows how molecule-in-cluster DFT-D restraints efficiently refine pharmaceutical crystal structures, outperforming earlier methods and guiding drug discovery pipelines	*Benchmarking quantum chemical methods with X-ray structures via structure-specific restraints* [Dittrich, B., Breznikar, R., Santarossa, G., Whitfield, P. & Moebitz, H. (2025). *IUCrJ*, **12**, 472–487]
Validating QM codes for QCr shows electron density depends on code and parameters, stressing convergence checks for reliable crystallographic analyses	*Code dependence of calculated crystalline electron densities. Possible lessons for quantum crystallography* [Landeros-Rivera, B., Contreras-García, J. & Martín Pendás, Á. (2025). *IUCrJ*, **12**, 295–306]
Benchmarking Hansen–Coppens multipolar refinement with DFT structure factors on copper acetate assesses the limits of parameterization and its alignment with quantum-mechanical accuracy	*On the flexibility of the multipole model refinement. A DFT benchmark study of the tetra­kis­(μ-acetato)di­aquadicopper model system* [Hlinčík, A., Fülöp, T., Herich, P., Kožíšek, J., Lušpai, K. & Bučinský, L. (2025). *IUCrJ*, **12**, 444–461]
Inverting X-ray restrained wavefunctions extracts perturbation potentials, showing how experimental data can guide improved DFT exchange–correlation functionals	*Inversion of the X-ray restrained wavefunction equations: a first step towards the development of exchange–correlation functionals based on X-ray data* [Genoni, A. & Sironi, M. (2025). *J. Appl. Cryst.***58**, 1106–1121]
QCr unites theory and experiment, combining scattering data to recover electron density matrices and revealing quantum behavior in crystals	*Intrusion of quantum crystallography into classical lands* [Yu, S. & Gillet, J.-M. (2025). *Acta Cryst.* B**81**, 168–180]. Commentary: *Pushing crystallography’s frontiers through quantum mechanics* [Matta, C. F. (2025). *Acta Cryst.* B**81**, 161–163]

**Table:** (*f*) Reviews

Summarizes 15 years of quantum crystallography advances in refinement methods, topological analyses and future experimental–computational developments	*Current developments and trends in quantum crystallography* [Krawczuk, A. & Genoni, A. (2024). *Acta Cryst.* B**80**, 249–274]
Review of thermal motion modeling, tracing the history of the Debye–Waller factor, ADP applications and advances, highlighting quantum crystallography’s role in refinement	*Models of thermal motion in small-molecule crystallography* [Hoser, A. & Madsen, A. Ø. (2025). *IUCrJ*, **12**, 421–434]. Commentary: *Everything you always wanted to know about the Debye–Waller factor but were afraid to ask* [Dronskowski, R. (2025). *IUCrJ*, **12**, 417–418]

## References

[bb1] Cooper, M. L. (2025). *IUCrJ* **12**, 614–615. 10.1107/S2052252524007164 PMC12573919 41143854

[bb2] Hoser, A. & Madsen, A. O. (2025). *IUCrJ* **12**, 421–434. 10.1107/S2052252525004361 PMC12224083 40476304

[bb4] Krawczuk, A. & Genoni, A. (2024). *Acta Cryst.* B**80**, 249–274. 10.1107/S2052520624003421 PMC11301899 38888407

[bb3] Massa, L., Huang, L. & Karle, J. (1995). *Int. J. Quantum Chem.* **56**, 371–384.

